# Quality of life, lifestyle, and working environment of women in the footwear industry

**DOI:** 10.47626/1679-4435-2020-517

**Published:** 2021-03-03

**Authors:** Lilian Rios, André Oliveira Paggiaro, Rosa Aurea Quintella Fernandes

**Affiliations:** Mestrado em Enfermagem, Programa de Pós-Graduação, Universidade Guarulhos, Guarulhos, SP, Brazil

**Keywords:** quality of life, working environment, lifestyle, occupational health

## Abstract

**Introduction:**

The footwear industry uses glue and other organic solvents (such as ethyl acetate, acetone, xylene, and toluene) in its manufacturing process; these substances, when associated with an inadequate working environment, can potentialize health problems and interfere with the perception of quality of life by female workers.

**Objectives:**

To verify the quality of life of women working in the footwear industry and its association with the self-reported morbidities of these workers, their working environment, and lifestyle habits.

**Methods:**

This is a cross-sectional study, with a quantitative approach, developed with 120 women shoemakers in the city of Franca, state of São Paulo. For evaluating quality of life, we used the abbreviated version of the World Health Organization Quality of Life instrument.

**Results:**

The most common self-reported morbidities were anxiety (65%), stress (62.5%), irritability (49.2%), sleep alterations (35.8%), fatigue (30%), and muscle cramps (30%). The physical domain presented the highest mean score (68.0), followed by the psychological (67.1) and social relationships domains (66.4). Environment was the domain with the lowest mean score (53.5). Quality of life was associated with the following self-reported morbidities: muscle cramps (p = 0.010), breathing difficulty (p = 0.029), tingling in the upper limbs (p = 0.010), decline in manual skills and work capacity, and pain (p < 0.001); the physical domain was the most affected. We observed a statistically significant difference in mean physical domain scores (p = 0.006) of women who used personal protection equipment; noise in the workplace interfered with the social relationships domain (p = 0.019), while working position interfered with the social relationships (p = 0.021) and environment (0 = 0.047) domains.

**Conclusions:**

The self-reported morbidities reported here and some working conditions negatively interfered with the women’s quality of life.

## INTRODUCTION

The footwear industry is an important sector of the Brazilian economy due to its production volume, important participation in the country’s exports, and capacity of generating jobs. Franca, a small city in the inland of the state of São Paulo, represents 2.81% of the country’s exports.^1^ In this municipality, the rate of women employed in stitching and gluing positions is approximately 55%.^2^

In Brazil, female work is generally characterized by higher precarity, lower salaries, and longer journeys when compared to that performed by men; these situations result in severe implications to the lives and health of female workers.^3^

The footwear industry uses, as raw materials, glue and organic solvents (including ethyl acetate, acetone, xylene, and toluene); these substances are potentially harmful to human health.^4^ Moreover, work in this sector involves other risks, such as noise, heat, vibration, humidity, extreme temperatures, small and poorly ventilated spaces, weight lifting, excessive work pace, repetition, inadequate posture, contact allergies, among other problems.^5,6^

Another frequently observed problem refers to the work environment. Women in the footwear industry are generally outsourced workers, whose salaries depend on the number of shoes they can produce. Therefore, workers subject themselves to long and uninterrupted working hours in inadequate environments. Unfortunately, this reality is not exclusive to the Brazilian female shoemakers: A cohort study with 138 Turkish shoemakers observed that they were subjected to long working hours in dangerous and inadequate conditions, which were worsened by the abuse of child labor and had neuropsychiatric effects on individuals.^7^

When considering the precarity of their working conditions and the specificities of their raw materials, Brazilian female shoemakers have an increased risk of developing health problems and diseases that can negatively influence their perceptions of quality of life (QoL). QoL is defined by the World Health Organization (WHO) as “individuals’ perceptions of their position in life in the context of the culture and value systems in which they live and in relation to their goals, expectations, standards, and concerns”. That is, QoL is a wide concept that involves aspects such as subjectivity, multidimensionality, and positive and negative dimensions.^8^

The working environment and lifestyle habits are believed to be related to the health problems reported by workers^9^ and, consequently, to a person’s own perception of his or her QoL. A recent study with workers of small and medium companies of 4 Asian countries concluded that healthy lifestyle habits, such as not smoking and exercising, in addition to reducing the workload and achieving adequate working conditions, improved the individuals’ QoL.^10^ In a cohort with 3357 Chinese drivers, worsening of QoL scores was associated with inadequate lifestyle habits, stressful working conditions, and comorbidities such as obesity.^11^

In view of the lack of studies and the situation of vulnerability at work faced by Brazilian female shoemakers, the objectives of this survey were to verify the QoL of women working in the footwear industry and study its association with self-reported morbidity, working environment, and lifestyle habits.

This knowledge should enable the creation of health actions aimed at this population, further allowing their QoL and health conditions to improve.

## METHODS

This is a cross-sectional, quantitative field survey performed with women shoemakers who worked at stitching centers in the municipality of Franca, state of São Paulo.

Our sample calculation considered the numbers presented by the General Registry of Employed and Unemployed Persons of the Ministry of Labor: 3425 women, with an allowable error of 3%, confidence interval of 95%, and standard deviation (SD) of 0.17; the minimum sample was thus defined as 120 women. Simple random sampling used the randomized.com website and a numerical list of 340 women who worked at stitching centers for large and medium shoe factories that agreed to provide their contact information. Women in our sample complied with the following inclusion criteria: being 18 years old or older and working in shoemaking for at least 1 year. Data were collected during the workers’ lunch hours in individual, previously scheduled interviews. A female surveyor guided the participants in filling the questionnaire and remained available for clarifications without influencing the answers. Data collection was performed in 2015, after project approval by the research ethics committee of Universidade Guarulhos under No. CAAE 39281314.0.0000.5506. All participants signed a free and informed consent form.

We used 2 instruments for data collection. The first questionnaire was the “social/labor and health profile of women shoemakers,” which included sociodemographic data such as age, marital status, ethnicity, religion, work position, family income, lifestyle habits (smoking and drinking habits, exercise, leisure activities), working conditions, and self-reported morbidity. For evaluating data related to work, we investigated the following aspects: ventilation, illumination, noise, use of protective equipment, evaluation of the workstation, and type of materials used in the work activity. For assessing self-reported morbidity, we elaborated a list of the most frequently observed health problems in this class of workers according to the literature; the interviewee indicated their presence or absence, as well as of other unrelated health problems, and evaluated their intensity and frequency of pain.

The second questionnaire was the abbreviated version of the World Health Organization Quality of Life instrument (WHOQOL-Bref),^12^ which allowed the measurement of the women’s QoL. This instrument consists in 26 questions and evaluates 5 domains: general, physical, psychological, social relationships, and environment. The answers are given in a Likert scale (1 to 5), and the score obtained by the sum of the participant’s answers can vary between 26 and 130; the higher the score, the better the QoL index.

Statistical analyses used an independent samples Student’s t-test and analysis of variance (ANOVA) for comparing means between 2 groups or between more than 2 groups, respectively. Both the Student’s t-test and ANOVA assumed that data is normally distributed, which was verified using a Kolmogorov-Smirnov test. In case the data were not normally distributed, nonparametric Mann-Whitney U tests (for comparing 2 means) and Kruskal-Wallis tests (for comparing more than 2 means) were used. The significance level was established as 5% for all tests.

## RESULTS

A total of 120 women working at stitching centers participated in this study. Their profile characteristics were: mean age 34.5 years (SD = 11.2), mostly White (51.7%), with complete secondary education (55%), catholic (57.5%), and performing household work with help (55.8%); 45.7% of them were married, 35.8% had one child, and their mean family income was US$ 658.00. As for their lifestyle habits, most of them were not tobacco (87.5%) or alcohol users (67.5%), they had some form of leisure activity (60%), and sought medical assistance when facing a health problem (90.8%). However, 60% of the women did not practice any kind of physical activity.

When considering aspects related to working conditions, all women in this study worked at formal stitching centers and considered them appropriate. The workstation was evaluated as good by 60.8% of workers, who mostly worked in a sitting position (60.8%). The occupations identified among shoemakers were gluing (53.3%), stitching (36.7%), folding (5.8%), and reviewing (4.2%). Most of the workers (78.3%) had been working in the same occupation for more than 4 years and worked in 2 shifts (95.8%), with a workday of 8 to 9 hours (97.5%). The most used materials were leather (100%), contact glue (95%), and solvents (61.7%). Most of the workers considered that the workplace had adequate illumination and ventilation (76.7% and 53.3%) and that the environmental noise was bearable or had a low intensity (65%). Personal protective equipment (PPE) was used by 78 workers (65%), and the most reported piece of equipment was hearing protection (64.7%).

Table 1 shows that the main self-reported morbidities were anxiety (65%), stress (62.5%), irritability (49.1%), sleep alterations (35.8%), fatigue and muscle cramps (30%), tingling or loss of sensation in the arms (26.7%), musculoskeletal pain (25%), and heat prostration (25%). The mean number of health problems per participant was 4.9 (SD = 3.4).

**Table 1:** Distribution of women according to self-reported morbidities, Franca, state of São Paulo, 2016 (n = 120)

Self-reported morbidity^*^	n	%
Anxiety	78	65.0
Stress	75	62.5
Irritability	59	49.2
Sleep alterations	43	35.8
Muscle cramps	36	30.0
Fatigue	36	30.0
Tingling of hands and forearm	32	26.7
Heat prostration	30	25.0
Musculoskeletal complaints	30	25.0
Ringing in the ears	26	21.7
Memory loss	25	20.8
Weakness	25	20.8
Breathing difficulty	23	19.2
Arterial hypertension	18	15.0
Temporary hearing loss	12	10.0
Decline in manual skills	11	9.2
Frequently purple and sweaty hands	7	5.8
Persistent hearing loss	7	5.8
Loss of conscience	6	5.0
Reduced work capacity	6	5.0
Others	1	0.8

* Multiple answers - the sum of all percentages does not equal 100%.

When investigating the use of medications and pain, we identified that 50.8% (n = 61) of the workers used medications, of which 85.2% had a medical prescription. Pain was reported by 75% of the workers; 67.8% reported feeling it always and 61.1% reported a disturbing pain intensity. The most frequently mentioned body areas were the shoulders/spine (47.8%).

Results of the QoL evaluation are shown in Table 2. The highest mean scores were reported in the physical domain (68.0), followed by the psychological (67.1) and social relationships domains (66.4). Environment presented the lowest mean score (53.5).

**Table 2:** Mean quality of life scores according to domains of the abbreviated version of the World Health Organization Quality of Life instrument (WHOQOL-Bref), Franca, state of São Paulo, 2016 (n = 120)

Domains	Mean	Standard deviation	Minimum	Maximum	Median
Physical	68.0	13.6	17.9	100.0	67.9
Psychological	67.1	16.6	12.5	95.8	70.8
Social relationships	66.4	20.0	16.7	100.0	66.7
Environment	53.5	14.5	12.5	96.9	56.3

When comparing mean QoL scores according to lifestyle habits, we observed a statistically significant difference only in the environment domain. Women who performed physical activity presented better QoL scores (p = 0.001).

The correlations between self-reported morbidity and QoL variables are shown in Table 3. Many of the reported health problems had a strong negative impact on QoL, especially on the physical domain. This influence was also observed on the psychological and environment domains, although less intensely. The least affected domain was social relationships, in which significant differences were only observed between women who mentioned ringing in the ears or not (p = 0.017). A statistically significant difference was observed between the QoL of workers who reported a health problem and those who did not. Specific problems that interfered with the physical domain of QoL were muscle cramps (p = 0.010), breathing difficulty (p = 0.029), tingling or loss of sensation in the upper limbs (p = 0.010), decline in manual skills and work capacity (p < 0.001), and pain (p < 0.001).

**Table 3:** Mean scores in the physical, psychological, social relationships, and environment domains compared to the self-reported morbidities of women in the footwear industry, Franca, state of São Paulo, 2016 (n = 120)

Self-reported morbidity	Physical domain		p-value	Psychological domain		p-value	Social relationships domain		p-value	Environment domain		p-value
	Mean	SD		Mean	SD		Mean	SD		Mean	SD	
Muscle cramps			0.010			0.652			0.668			0.969
No	70.10	13.10		66.70	16.70		65.90	20.10		53.50	14.50	
Yes	63.10	13.80		68.20	16.70		67.60	20.00		53.40	14.70	
Respiratory difficulty			0.029			0.341			0.647			0.383
No	69.30	12.90		67.80	16.60		66.00	19.60		54.00	14.60	
Yes	62.40	15.70		64.10	16.70		68.10	22.10		51.10	14.10	
Hearing loss			0.007			0.009			0.293			0.019
No	68.80	13.00		68.10	15.90		65.90	19.70		54.20	14.00	
Yes	54.60	18.10		51.20	21.70		73.80	24.70		41.10	17.20	
Temporary hearing loss			0.222			0.031			0.791			0.046
No	68.50	13.80		68.20	15.60		66.20	20.20		54.30	13.70	
Yes	63.40	12.00		57.30	22.60		68.10	18.70		45.60	19.10	
Stress			< 0.001			0.001			0.126			0.007
No	73.90	12.20		73.10	12.50		70.00	18.80		58.10	12.30	
Yes	64.40	13.30		63.60	17.90		64.20	20.50		50.70	15.00	
Memory loss			0.030			0.140			0.653			0.010
No	69.40	13.20		68.40	15.80		66.00	20.10		55.20	13.90	
Yes	62.70	14.40		62.20	19.20		68.00	19.90		46.90	14.90	
Tingling, loss of sensation in the upper limbs			0.010			0.819			0.860			0.175
No	69.90	12.60		67.30	16.80		66.20	19.90		54.50	15.00	
Yes	62.70	15.30		66.50	16.30		66.90	20.70		50.50	12.80	
Weakness			0.002			0.349			0.467			0.010
No	69.90	13.10		67.90	17.30		67.20	19.40		55.20	13.70	
Yes	60.70	13.60		64.30	13.90		63.30	22.40		46.90	15.60	
Irritability			0.016			0.006			0.823			0.051
No	70.90	13.30		71.20	13.60		66.00	20.10		56.00	13.70	
Yes	65.00	13.40		62.90	18.50		66.80	20.00		50.80	14.90	
Heat prostration			< 0.001			0.049			0.861			0.109
No	70.50	12.40		68.80	16.50		66.20	20.70		54.70	14.00	
Yes	60.50	14.70		61.90	16.20		66.90	18.10		49.80	13.00	
Decline in manual skills			< 0.001			0.825			0.135			0.270
No	69.40	12.60		67.00	17.00		65.50	20.10		53.90	14.80	
Yes	54.20	16.20		68.20	12.90		75.00	17.90		48.90	10.60	
Reduced work capacity			< 0.001			0.937			0.618			0.812
No	69.20	12.60		67.00	16.80		66.20	20.00		53.50	14.70	
Yes	45.20	14.80		68.80	14.10		70.80	21.60		52.10	10.60	
Pain			< 0.001			0.299			1.000			0.421
No	79.40	11.60		66.20	16.30		66.40	19.40		52.80	14.80	
Yes	64.20	12.10		69.90	17.70		66.40	20.30		55.30	13.50	
Ringing in the ears			0.064			0.061			0.017			0.733
No	69.20	13.10		66.00	17.80		64.50	20.90		53.20	14.20	
Yes	63.60	15.00		71.30	10.90		73.40	14.90		54.30	15.60	

SD = standard deviation.

Persistent hearing loss altered the physical (p = 0.007), psychological (p = 0.009), and environment (p = 0.019) domains. Temporary hearing loss interfered with the psychological (p = 0.031) and environment (p = 0.046) domains. Stress and irritability modified the physical (p < 0.001 and p = 0.016), psychological (p < 0.001 and p = 0.006), and environment (p = 0.007 and p = 0.051) domains. Weakness altered the physical (p = 0.002) and environment (p = 0.010) domains, while heat prostration affected the physical (p < 0.001) and psychological (p = 0.049) domains.

Table 4 indicates a statistically significant difference between mean QoL scores of the physical domain (p = 0.006) for women who used PPE in relation to those who did not; workers who used protective equipment had better QoL scores. Environmental noise interfered with the QoL score of the social relationships domain (p = 0.019), while working position affected the social relationships (p = 0.021) and environment (p = 0.047) domains.

**Table 4:** Mean scores in the physical, psychological, social relationships, and environment domains compared to work and work environment characteristics, Franca, state of São Paulo, 2016 (n = 120)

Characteristics and work environment	Physical domain		p-value	Psychological domain		p-value	Social relationships domain		p-value	Environment domain		p-value
	Mean	SD		Mean	SD		Mean	SD		Mean	SD	
Use of personal protective equipment			0.006			0.430			0.920			0.920
Yes	70.50	12.40		68.00	17.80		66.90	19.00		53.40	14.90	
No	63.40	14.80		65.50	14.40		65.50	22.00		53.60	13.70	
Environmental noise			0.214			0.908			0.019			0.478
None	85.70			70.80			58.30			59.40		
Little	69.10	17.90		69.00	45.80		74.70	25.00		56.90	25.00	
Bearable	69.40	42.90		66.10	25.00		62.30	16.70		53.40	21.90	
Intense	64.80	42.90		67.20	12.50		67.10	25.00		51.20	12.50	
Working position			0.135			0.512			0.021			0.047
Standing	65.70	13.20		65.90	14.80		61.20	20.40		50.20	14.40	
Sitting	69.50	13.80		67.90	17.80		69.70	19.20		55.60	14.20	

SD = standard deviation.

## DISCUSSION

The complexity of the work process and the increase in time invested in labor activities act dynamically together and with the worker’s body, implicating in wear and tear processes that can cause acute or chronic diseases, unspecific signs and symptoms, or premature aging.^13^

Professional work, when associated with other activities performed by women, can prolong their daily workload to up to 12 hours, which could contribute to a higher incidence of accidents and health problems that compromise women’s health.^14^

In this survey, the studied sample predominantly consisted of young women (mean age 34.5 years), with an intermediate level of education and who accumulated professional activities and household work. These women had relatively healthy lifestyle habits, since most of them did not present tobacco or alcohol use; however, 60% did not perform any type of physical activity. Various studies have demonstrated that physical activity improves QoL in individuals with chronic pain or depression, especially in the physical and psychological domains.^15,16^

When considering working conditions, some women worked in conditions that can be considered precarious: 39.2% evaluated their workstations as inadequate or worked standing up; 46.7% worked with inadequate ventilation even though almost all workers were exposed to toxic substances (glue and solvents); and 32.5% considered the environmental noise to be intense. These data indicate that production working conditions are far from ideal. A study performed with 16 926 footwear industry workers in Colorado, USA, demonstrated that absenteeism and low productivity, as well as chronic diseases developed by the workers, were intimately linked to unsafe working environments.^17^

Stress, anxiety, irritability, sleep alterations, fatigue, pain in the shoulders and/or spine, ringing in the ears, and musculoskeletal complaints were some of the self-reported morbidities of women shoemakers. Most of them (75%) mentioned pain. Pain exerts a negative influence on work quality, resulting in a high cost for the society and a great impact on the individual’s QoL, since it leads to losses in production time and to suffering and limitations in daily life.^18^

A study performed in Sobral, in the state of Ceará, showed that many activities performed in shoe factories caused serious health problems due to the production line and/or conveyor belt work.^19^ In the gluing sector, workers complained of migraine, dizziness, and nausea caused by the direct manipulation of paint and solvents, which are toxic. Female workers of the textile industry in the Northwest region of the state of Paraná also presented pain related to repetitive movements (12.4%) and back pain (34.8%), which corroborates the present results.^20^

Regarding QoL analysis, to the best of our knowledge, no studies have evaluated this aspect in workers of the footwear industry. In a study performed with workers of small food and textile industries of 4 Asian countries, using the same survey instrument as this study and in which most participants were female, QoL scores were higher in most domains when compared to Brazilian workers. Only the psychological (67.4) and social relationships (63.6) domain scores of Indonesian workers were similar to the ones obtained in this study. Indonesia suffers with unemployment, and most workers accept longer working hours in order to maintain their employment bonds; however, this reflects in a worsening of QoL. Brazilian shoemakers also suffer with long workloads both in the industry and in the household, thus presenting inferior results in all domains, similarly to Indonesian workers.^10^

Another study performed in a Brazilian company that produces school supplies, considering 100 individuals who were mostly female and with a mean age of 34.8 years, observed the following scores: 69.40 in the physical domain, 68.91 in the psychological domain, 71.96 in social relationships, and 55.79 in the environmental domain.^21^ These results are similar to the ones obtained in this study.

When considering the correlation between lifestyle habits and QoL, physical activity was the only aspect that positively affected QoL perception. The benefits of physical exercise are known in improving cardiovascular fitness and preventing diseases. In our study, we observed an improvement in the environmental domain (p = 0.001), which assesses physical safety and security, home environment, financial resources, health and social care, opportunities for acquiring new information and skills, recreation/leisure, physical environment (pollution/noise/traffic/climate), and transport. This result is in line with those reported by a group of researchers from Taiwan, who evaluated the relationship between weight loss and QoL in a cohort of 67 overweight individuals. In this study, participants underwent a diet and physical exercise program. Those who managed to complete the program, with weight loss and physical exercise, presented improvements in global QoL, especially in the environment domain (p = 0.002).^22^

As for work conditions, not all women in our sample used PPE, and those who did not use it had lower QoL scores, especially in the physical domain. A survey that analyzed the impact of environmental noise on QoL verified that not all employees used PPE, and noise interfered negatively with QoL; thus, prevention and orientation programs on the importance of PPE were suggested.^23^ Noise was also associated with a lower QoL score in the environmental domain in a multi-center study.^10^

Various self-reported morbidities negatively influenced the QoL scores in this study. Muscle cramps (p = 0.010), breathing difficulty (p = 0.029), tingling or loss of sensation in the upper limbs (p = 0.010), and pain (p < 0.001) were the health problems that only altered the physical domain score. Stress and irritability interfered with the physical (p < 0.001 and p = 0.016), psychological (p < 0.001 and p = 0.006), and environmental (p = 0.007 and p = 0.051) domains. A qualitative survey evaluating QoL at work and its repercussions in health identified that workloads generate stress and body pain.^24^ Another study indicated that women with more precarious jobs suffered more from stress.^25^ Persistent and temporary hearing loss caused alterations in various domains of QoL, which reinforces the need to stimulate the use of PPE. The generalization of the results of this study should be limited to developing countries, where working conditions still need to be better established and monitored by government control agencies. An important limitation of this study lies in the fact that our participants were only women working at formal stitching centers. However, many other women work in home-based stitching centers, with no minimal occupational safety control. This leads us to believe that QoL indices would be worse if these women working in much more precarious conditions were added to the study.

As implications for practice, this study contributes to the elaboration of health actions aimed at this population. Prevention programs could improve the health of these women; the number of women shoemakers in Brazil is relevant and they deserve a more accurate look to the repercussions of their work in their health.

## CONCLUSIONS

The QoL of women in the footwear industry is similar to that of other professionals. Self-reported morbidities negatively interfere with their QoL, since a statistically significant difference was observed between those who reported health problems and those who did not. The physical domain was the most affected. We observed few influences of lifestyle habits on QoL, but the work environment had a negative effect. It is fundamental that footwear industries not only provide PPE for these women, but also monitor their use and guarantee periodic medical examinations focusing on the health problems caused by their professional activity. Health promotion actions could contribute to improve the health and QoL of these workers.
